# Quantitative analysis of pathogens in the lower respiratory tract of patients with chronic obstructive pulmonary disease

**DOI:** 10.1186/s12890-015-0094-z

**Published:** 2015-08-19

**Authors:** Huaying Wang, Xiao Gu, Yuesong Weng, Tao Xu, Zhongming Fu, Weidong Peng, Wanjun Yu

**Affiliations:** Department of Respiratory Diseases, Affiliated Yinzhou Hospital, College of Medicine, Ningbo University, 251 East Baizhang Road, Ningbo City, Zhejiang Province 315040 P. R. China; Clinical Laboratory, First Hospital, 58 Liuting Road, Ningbo City, Zhejiang Province 315010 P. R. China

## Abstract

**Background:**

Bacterial infection of the lower respiratory tract is believed to play a major role in the pathogenesis of chronic obstructive pulmonary disease (COPD) and acute exacerbations of COPD (AECOPD). This study investigates the potential relationship between AECOPD and the load of six common bacterial pathogens in the lower respiratory tract using real-time quantitative PCR (RT-qPCR) in COPD patients.

**Methods:**

Protected specimen brush (PSB) and bronchoalveolar lavage fluid (BALF) samples from the lower respiratory tract of 66 COPD patients and 33 healthy subjects were collected by bronchoscopy. The load of *Staphylococcus aureus*, *Klebsiella pneumoniae*, *Streptococcus pneumoniae*, *Pseudomonos aeruginosa*, *Haemophilus influenzeae*, and *Moraxella catarrhalis* were detected by RT-qPCR.

**Results:**

High *Klebsiella pneumoniae*, *Pseudomonos aeruginosa*, *Haemophilus influenzeae* and *Moraxella catarrhalis* burden were detected by RT-qPCR in both PSB and BALF samples obtained from stable COPD and AECOPD patients compared with healthy subjects. The load of the above four pathogenic strains in PSB and BALF samples obtained from AECOPD patients were significantly higher compared with stable COPD patients. Finally, positive correlations between bacterial loads and inflammatory mediators such as neutrophil count and cytokine levels of IL-1β, IL-6 and IL-8, as well as negative correlations between bacterial loads and the forced expiratory volume in one second (FEV1) % predicted, forced vital capacity (FVC) % predicted, and FEV1/FVC ratio, were detected.

**Conclusions:**

These findings suggest that increased bacterial loads mediated inflammatory response in the lower respiratory tract and were associated with AECOPD. In addition, these results provide guidance for antibiotic therapy of AECOPD patients.

## Background

Progressive and irreversible airflow obstruction are characteristics of chronic obstructive pulmonary disease (COPD), which are caused by chronic inflammation of airways [1]. COPD is a major cause of morbidity and mortality worldwide and is projected to become the third leading cause of death by 2030 [2]. The major therapeutic goal of COPD treatment is to prevent acute exacerbations. However, available treatments for acute exacerbations of COPD (AECOPD) are currently not very effective [3]. Bacterial infections of the lower respiratory tract are believed to play a major role in the pathogenesis of AECOPD. Generally, nontypeable *Haemophilus influenzae*, *Streptococcus pneumoniae*, and to a lesser extent, *Moraxella catarrhalis* are the most frequent species isolated by microbiological culture during COPD exacerbations [4]. These organisms can often be found colonizing the airways of COPD patients between exacerbations [5]. Since many of these bacteria persist in the airways of COPD patients, their presence may promote chronic inflammatory states that drives COPD pathogenesis. However, information on the quantitative analysis of bacterial burden in the lower respiratory tract in stable COPD and AECOPD patients are quite limited. Previous studies frequently analyzed bacteria in the respiratory tract of patients by traditional microbiological culture techniques. However, the current microbiological gold standard has a number of limitations, particularly the lack of sensitivity and time-consuming culture; which significantly impacts the treatment and management of patients, and limits our understanding of the development and progression of COPD [4]. Furthermore, bacterial cultures can lead to false-negative results, especially during concurrent antibiotic treatment. A challenge in COPD diagnostics is to distinguish disease-causing strains from colonizing strains. It has been shown that for *Streptococcus pneumoniae* [6], the load of many types of bacteria in the respiratory tract is probably greater during infection than during carriage; and therefore, quantitative methods would most likely improve diagnostic quality.

In recent years, real-time quantitative PCR (RT-qPCR) has emerged as a valuable tool for the quantitative and rapid detection of various biological specimens in body fluids [7, 8]. Guma *et al*. developed a RT-qPCR method using primers and a TaqMan probe complementary to sequences in the omp P6 gene for rapid detection of *Haemophilus influenzeae*, and their study concluded that P6 RT-qPCR is both sensitive and specific for identifying *Haemophilus influenzeae* in respiratory secretions [9]. In another study, the prevelance of *Moraxella catarrhalis* was detected by RT-qPCR using primers and probes targeting the copB gene, which provided a sensitive and reliable means of rapidly detecting and quantifying *Moraxella catarrhalis* during lower respiratory tract infections; and this may be applied to other clinical samples [10]. In addition, some other common pathogenic bacteria in respiratory tract infections such as *Klebsiella pneumoniae* [11], *Staphylococcus aureus* [12], *Streptococcus pneumoniae* [13], and *Pseudomonos aeruginosa* [14] were detected by RT-qPCR, targeting bacteria-specific genes. These methods present sensitive and reliable means of rapidly detecting and quantifying microorganisms. The load of common bacteria in the respiratory tract of patients with COPD or lower respiratory tract infection was analyzed by RT-qPCR or multiplex PCR in sputum samples [4, 15]^.^ In another study, the spectrum of potentially pathogenic microorganisms in sputum of COPD patients was determined by PCR-denaturing gradient gel electrophoresis (DGGE) [16]. However, most of these previous studies focused on the respiratory tract microbiome of COPD patients in the stable stage [17], and only few studies compared common bacteria between the stable stage and acute exacerbations of COPD. Furthermore, most of the studied samples were sputum or bronchoalveolar lavage fluid (BALF) samples, in which contamination by pathogens colonizing the upper respiratory tract are difficult to avoid. Therefore, proper collection of samples from the lower respiratory tract is a key for the precise quantitative analysis of pathogens. The usefulness of a protected specimen brush (PSB) for diagnosing respiratory infections has been reported earlier [18–20]. The tip of the sampling brush or PSB is covered by a sheath to avoid contamination by organisms in the upper tract while the brush is being inserted or pulled out [21]. Thus, PSB or protected BALF from bronchoscopy appears as the best choice for sample collection from the lower respiratory tract in COPD patients.

In this present study, we describe the application of RT-qPCR in tageting specific bacterial pathogen genes for the simultaneous and direct detection and quantification of a range of the most common pathogens in the lower respiratory tract including Staphylococcus aureus, Klebsiella pneumoniae, Streptococcus pneumoniae, Pseudomonos aeruginosa, Haemophilus influenzeae, and Moraxella catarrhalis in PSB and BALF samples obtained from stable COPD and AECOPD patients. Moreover, we explored the relationship among bacterial burden, inflammtory response such as neurotophil count and cytokine levels of IL-1β, IL-6 and IL-8 in BALF, and the forced expiratory volume in one second (FEV1) % predicted, forced vital capacity (FVC) % predicted, and FEV1/FVC lung function values in COPD patients. Our study is the first to describe common bacteria in the lower respiratory tract by RT-qPCR analysis using PSB and protected BALF samples, and compare the change of bacterial load between the stable stage and acute exacerbations of COPD. These results may provide guidance for the effective and timely antibiotic treatment of AECOPD patients.

## Materials and Methods

### Study subjects

Sixty-six COPD patients (GOLD stage II-III, COPD group) and 33 healthy subjects (HS group) with normal pulmonary function (non-smokers) were enrolled in this case–control study, which was carried out at the Department of Respiratory Diseases, Affiliated Yinzhou Hospital, College of Medicine, Ningbo University, China. COPD was diagnosed according to the patient’s history of tobacco smoking, symptoms, and post-bronchodilator pulmonary function tests with FEV1/FVC lower than 70 %, according to Global Initiative for Chronic Obstructive Lung Disease (GOLD) guidelines [22]. All patients were clinically assessed including chest radiography, temperature recording and blood gas analysis (as needed) to exclude other causes of breathlessness. Moreover, patients in the COPD group were further divided into two subgroups according to disease stage: stable COPD group and AECOPD group. The stable COPD group consisted of 34 patients who were evaluated as clinically stable and underwent an exacerbation-free period of six weeks. Patients in the stable COPD and HS groups were examined by bronchoscopy to exclude other lung lesions. The AECOPD group consisted of 32 patients who were identified according to the Anthonisen criteria [23] and the consensus definition for COPD exacerbations [24]. Patients in the AECOPD group were diagnosed according to pulmonary function and underwent mechanical ventilation via tracheal tubing to treat type-II respiratory failure. Bronchoscope examinations were guided by a tracheal tube. All subjects enrolled in this study underwent routine blood examination. Patients that presented with pneumonia, neuromuscular diseases, thoracic deformities, restrictive lung diseases, pulmonary vascular disease, as well as patients who underwent lung resection, were excluded from this study. Two hours after tracheal tube intubation and before antibiotic treatment, lower respiratory tract samples were collected by a protected specimen brush (PSB) and protected bronchoalveolar lavage (BAL) during bronchoscopy. This study protocol was authorized by the ethics and research committee of the Affiliated Yinzhou Hospital, College of Medicine, Ningbo University, China. Signed informed consent was obtained from all subjects.

### Collection of PSB and BALF samples from patients

PSB samples were collected with a sheath brush (Olympus BC-5 CE, Olympus Imaging, Center Valley, PA, USA) with a distal occlusion composed of polyethylene glycerol through a flexible bronchoscope (Olympus, BF-260). The brush was pushed out of the sheath, cut with ethanol-disinfected scissors, and placed in an Eppendorf tube containing 1.5 ml of saline solution. A part of the PSB samples (0.5 ml) were used for standard bacterial cultures and remaining samples were prepared for total DNA extraction. Simultaneously with PSB sample collection, BAL specimens were obtained by lavaging the airway with approximately 50 ml of 0.9 % NaCl solution through the bronchoscope; and approximately 60 % lavage return volume was collected. Total BALF cells were counted from a 0.05 ml aliquot, 0.5 ml of BALF samples were used for bacterial cultures, and 1 ml of BALF samples were taken for total DNA extraction. Remaining fluid samples were centrifuged (1,000 g for 10 min) at 4 °C, and the supernatant was stored at −80 °C for subsequent cytokine analysis by ELISA. The remaining cell pellets were resuspended with 0.9 % NaCl solution, and a differential cell count was performed using cytospin and Wright-Giemsa staining.

### Bacterial cultures

PSB and BALF samples were inoculated into Eosin methylene blue agar, Brucella agar, blood agar, and chocolate agar media (Biomérieux, Marcy l'Etoile. France); and incubated for 24–48 h at 37 °C. Colony identification was performed using the Vitek 2 Compact full automatic identification system (Biomérieux, France). For PSB and BALF cultures, after 24 h of incubation, bacterial colonies with growth of ≥10^3^ and ≥10^5^, respectively, were considered as pathogenic.

### Cytokine ELISA

IL-1β, IL-6, IL-8, IL-10 and TNF-α cytokine concentrations in BALF supernatants were measured by standardized sandwich ELISA (eBioscience, San Diego, CA, USA) according to manufacturer’s protocols.

### Preparation of bacterial DNA from PSB and BALF samples

PSB or BALF samples (1 ml) were centrifuged at 11,000 g for five minutes. Aliquots of 0.2 ml of the pellet were used in order to obtain a 5-fold concentration of the samples. DNA was prepared using a Qiagen DNA Mini Kit (Qiagen, Hilden, Germany) according to manufacturer’s instructions. DNA was stored at −20 °C before amplification by RT-qPCR. In each experiment, a negative control that contained all reagents except the PSB sample was included.

### RT- qPCR of PSB and BALF samples

Primers and TaqMan probes for the specific amplification of *Staphylococcus aureus*, *Klebsiella pneumoniae*, *Streptococcus pneumoniae*, *Pseudomonos aeruginosa*, *Haemophilus influenzeae* and *Moraxella catarrhalis* were synthesized by Sangon Biotech Co., Ltd. (Shanghai, P.R. China). Primer sequences and assay performance are summarized in Table 1. DNA amplification and detection were performed with a TaqMan 7500 Fast system (Applied Biosystems, Foster City, CA, USA). The reaction mixture (20 μl) used in the PCR assay was as follows: 10 μl of TaqMan Universal PCR Mastermix (Applied Biosystems), 2 μl of extracted DNA, 0.5 μl of specific primers (final concentration was 0.6 μmol/l), and probes (final concentration was 0.3 μmol/l). PCR cycling conditions applied for khe, cps, omp P6, and copB gene assays were as follows: heating at 94 °C for four minutes, followed by 40 cycles of 94 °C for 30 s and 60 °C for one minute. PCR cycling conditions applied for egc and gyrB gene assays were as follows: heating at 95 °C for 10 min, followed by 45 cycles of 95 °C for 15 s and 60 °C for one minute. Optimized conditions of the RT-qPCR assay are summarized in Table 2. Fluorescence was measured after each cycle, and each assay was carried out in duplicate. Two negative RT-qPCR controls were run on each sample plate, and the median cycle of quantification (Cq) value from each duplicate was used for analysis.

**Table 1:** RT-qPCR primers and assay performance summary

Organism	Target gene	Primer/Probe sequences	Assay linearity	Ref.
*Staphylococcus aureus*	*egc*	F:5′-CTTCATATGTGTTAAGTCTTGCAGCTT-3′	R^2^ = 0.995	[12]
		R:5′-TTCACTCGCTTTATTCAATTGTTCTG-3′	Slope = −3.91	
		P: 5′-6-FAM -ATGTTAAATGGCAATCCT-TAMRA -3′	Efficiency = 1.04	
*Klebsiella pneumoniae*	*khe*	F: 5′-GATGAAACGACCTGATTGCATTC-3′	R^2^ = 0.997	[11]
		R: 5′-CCGGGCTGTCGGGATAAG-3′	Slope = −3.60	
		P:5′-6-FAM-CGCGAACTGGAAGGGCCCG-TAMRA-3′	Efficiency = 1.07	
*Streptococcus pneumoniae*	*cps*	F: 5′-GCTGTTTTAGCAGATAGTGAGATCGA-3′	R^2^ = 0.995	[13]
		R: 5′-TCCCAGTCGGTGCTGTCA-3′	Slope = −3.11	
		P: 5′-6-FAM-AATGTTACGCAACTGACGAG-TAMRA -3′	Efficiency = 1.12	
*Pseudomonos aeruginosa*	*gyrB*	F: 5′-GGCGTGGGTGTGGAAGTC-3′	R^2^ = 0.995	[14]
		R: 5′-TGGTGGCGATCTTGAACTTCTT-3′	Slope = −3.55	
		P: 5′-6-FAM-TGCAGTGGAACGACA-TAMRA-3′	Efficiency = 0.96	
*Haemophilus influenzeae*	*omp P6*	F:5′-CCAGCTGCTAAAGTATTAGTAGAAG-3′	R^2^ = 0.997	[9]
		R: 5′-TTCACCGTAAGATACTGTGCC-3′	Slope = −4.22	
		P: 5′-6-FAM -CAGATGCAGTTGAAGGTTATTTAG-	Efficiency = 0.95	
		TAMRA-3′		
*Moraxella catarrhalis*	*copB*	F: 5′-GTGAGTGCCGCTTTACAACC-3′	R^2^ = 0.998	[10]
		R: 5′-TGTATCGCCTGCCAAGACAA-3′	Slope = −3.66	
		P:5′-6-FAM-TGCTTTTGCAGCTGTTAGCCAGCCTAA-	Efficiency = 0.91	
		TAMRA-3′

**Table 2:** Optimized conditions for egc, khe, cps, gyrB, omp P6 and copB RT-qPCR assay

Reaction	Final concentration					
Components	*egc*	*khe*	*cps*	*gyrB*	*omp P6*	*copB*
TaqMan Universal PCR Mastermix	1×	1×	1×	1×	1×	1×
Primer	0.6 μmol/L	0.6 μmol/L	0.6 μmol/L	0.6 μmol/L	0.6 μmol/L	0.6 μmol/L
Probe	0.3 μmol/L	0.3 μmol/L	0.3 μmol/L	0.3 μmol/L	0.3 μmol/L	0.3 μmol/L
Total volume	20 μl	20 μl	20 μl	20 μl	20 μl	20 μl
Melt	95 °C, 10 min	94 °C, 4 min	94 °C, 4 min	95 °C, 10 min	94 °C, 4 min	94 °C, 4 min
Denaturation	95 °C, 15 s	94 °C, 30 s	94 °C, 30 s	95 °C, 15 s	94 °C, 30 s	94 °C, 30 s
Annealing/Extension	60 °C, 60 s	60 °C, 60 s	60 °C, 60 s	60 °C, 60 s	60 °C, 60 s	60 °C, 60 s
Cycles	45	40	40	45	40	40
PCR Product Size	82 base pairs	77 base pairs	67 base pairs	190 base pairs	156 base pairs	71base pairs

**Table 3:** Demographic and clinical characteristics of patients in the HS, stable COPD and AECOPD groups

	HS	Stable COPD patients	AECOPD patients
	(*n* = 33)	(*n* = 34)	(*n* = 32)
Age (years)	65 ± 8	67 ± 7	69 ± 6
Gender (M/F)	20/13	28/6	27/5
BMI (kg/m^2^)	23.1 ± 5.1	24.2 ± 4.9	23.8 ± 3.9
Tobacco (pack/year)	-	44.2 ± 25.6	43.7 ± 27.8
FEV1 (L)	3.3 ± 0.3	1.3 ± 0.1^**^	1.2 ± 0.2^**^
FEV1 % predicted	103.6 ± 7.3	53.4 ± 8.8^**^	51.4 ± 9.0^**^
FVC (L)	4.0 ± 0.2	2.9 ± 0.2^*^	2.7 ± 0.2^*^
FVC % predicted	102.7 ± 6.7	81.4 ± 9.3^**^	79.9 ± 7.4^**^
FEV1/FVC (%)	82.3 ± 6.1	43.8 ± 4.4^**^	44.1 ± 7.1^**^
WBC (x10^9^/L)	5.6 ± 1.2	6.1 ± 1.7	11.4 ± 5.1^**##^
Neutrophils (%)	64.6 ± 6.7	68.7 ± 7.6^*^	85.4 ± 6.1^**##^
CRP (mg/L)	6.7 ± 2.4	16.1 ± 6.7^**^	85.2 ± 30.4^**##^

All data are expressed as means ± SEM, except gender. ^*^ *P* < 0.05 and ^**^ *P* < 0.01 *vs*. the HS group; ^#^ *P* < 0.05 and ^##^ *P* < 0.01 *vs*. the stable COPD group; FEV1, Forced expiratory volume in one second; FVC, forced vital capacity; CRP, C-reactive protein; HS, Healthy subjects. The spirometry data in AECOPD patients reflect the baseline data in the stable stage

**Table 4:** Microbiological culture results of PSB and BALF samples obtained from patients in the HS, stable COPD and AECOPD groups

*n* (%)	HS		Stable COPD patients		AECOPD patients	
	(*n* = 33)		(*n* = 34)		(*n* = 32)	
	PSB	BALF	PSB	BALF	PSB	BALF
*No isolated or Normal flora*	29 (87.9)	29 (87.9)	20 (58.8)	15 (44.1)	9 (28.1)	8 (25.0)
*Staphylococcus aureus*	1 (3.0)	1 (3.0)	2 (5.9)	4 (11.8)	3 (9.4)	3 (9.4)
*Klebsiella pneumoniae*	1 (3.0)	1 (3.0)	1 (2.9)	3 (8.8)	4 (12.5)	4 (12.5)
*Streptococcus pneumoniae*	2 (6.0)	2 (6.0)	4 (11.8)	5 (14.7)	3 (9.4)	4 (12.5)
*Pseudomonos aeruginosa*	0	0	2 (5.9)	2 (2.9)	4 (12.5)	5 (15.6)
*Haemophilus influenzeae*	0	0	2 (5.9)	3 (8.8)	6 (18.8)	5 (15.6)
*Moraxella catarrhalis*	0	0	1 (2.9)	2 (2.9)	2 (6.3)	2 (6.3)
*Others* ^a^	0	0	1 (2.9)	2 (2.9)	1 (3.1)	1 (3.1)

^a^Other isolated bacteria including *Acinetobacter baumanii*, *Escherichia coli*, and *Streptococcus hemolyticus*. HS, Healthy subjects

### Quantification of microorganism load in PSB and BALF samples

To quantify the number of bacterial cells in PSB and protected BALF samples, six ATCC standard strains were used as positive controls. *Staphylococcus aureus* (ATCC 25923) and *Pseudomonos aeruginosa* (ATCC 27853) were provided by the Clinical Laboratory, First Hospital, Ningbo City, China. *Klebsiella pneumoniae* (ATCC 700603), *Streptococcus pneumoniae* (ATCC 49619), *Haemophilus influenzeae* (ATCC 49247), and *Moraxella catarrhalis* (ATCC 25238) were purchased from Bioplus Biotech Co., Ltd. (Shanghai, China). A dense suspension of bacteria grown on agar plates was inoculated in phosphate-buffered saline, representing a bacterial concentration of approximately 10^8^ CFU/ml. A 10-fold serial dilution scheme ranging between 10^8^ and 10^3^ CFU/ml was prepared. To correlate cycle threshold (CT) values measured by RT-qPCR with the number of bacterial cells present in each sample, aliquots (100 ml) of each dilution of bacterial suspension were plated out in triplicate onto agar plates. Agar plates were incubated overnight at 37 °C, and colonies were counted in order to calculate the number of CFU per dilution tube. DNA extraction was performed from a sample (5 ml) of each dilution tube and was analyzed concomitantly by RT-qPCR. Each positive control was carried out in triplicate, mean CT values were calculated and plotted against the base 10 logarithm of CFU per ml, and a standard curve was generated. The load of microorganisms in PSB and BALF samples was determined using a standard equation.

### Statistical analysis

All data were expressed as means ± SEM. Differences between groups were examined for statistical significance by one-way analysis of variance (ANOVA) using SPSS 11.0 software (SPSS Inc., Chicago, USA). The correlation among bacterial burden, inflammatory mediators such as neutrophil cell count and cytokine levels, and pulmonary function such as FEV1 % predicted, FVC % predicted, and FEV1/FVC were calculated using *Pearson*’s correlation coefficient. *P* values <0.05 denoted that the difference was statistically significant.

## Results

### Demographic and clinical features and pulmonary function of COPD patients and healthy subjects (HS)

Demographic and clinical features, spirometric findings, and routine blood examination results of patients in the HS, stable COPD and AECOPD groups are shown in Table 3. Age, gender, and BMI did not differ among the three groups. Spirometry, FVC, FVC % predicted, FEV1, and FEV1 % predicted significantly decreased in patients in the stable COPD group compared with patients in the HS group (*P* < 0.05). However, the percentage of neutrophils and C-reactive protein (CRP) levels were significantly higher in patients in the stable COPD and AECOPD groups compared with patients in the HS group (*P* < 0.05), and between patients in the stable COPD and AECOPD groups (*P* < 0.01). White blood cell count significantly increased in patients in the AECOPD group compared with patients in the stable COPD and HS groups (*P* < 0.01). However, there was no significant difference between patients in the stable COPD and HS groups (*P* > 0.05).

### Microbiological cultures from PSB and BALF samples obtained from COPD patients and HS

PSB and BALF samples were obtained through a flexible bronchoscope to analyze bacteria in the lower respiratory tract of COPD patients and HS. Microbiological cultures revealed that 29 HS (87.9 %) had no isolated or normal non-pathogenic bacterial flora in PSB and BALF samples (Table 4). In 20 (58.8 %) and 15 COPD patients (44.1 %), no isolated or normal airway bacterial flora was detected in PSB and BALF samples, respectively. In nine (28.1 %) and eight (25.0 %) AECOPD patients, no isolated or normal airway bacterial flora was detected in PSB and BALF samples, respectively. The following pathogenic bacteria were identified in cultures of the remaining patients: *Staphylococcus aureus*, *Klebsiella pneumoniae*, *Streptococcus pneumoniae*, *Pseudomonos aeruginosa*, *Haemophilus influenzeae*, and *Moraxella catarrhalis*. Other isolated bacteria included *Acihetobacter baumanii*, *Escherichia coli*, and *Streptococcus hemolyticus* (Table 4).

### RT-qPCR analysis of bacteria in PSB and BALF samples obtained from COPD patients and HS

Total bacterial DNA in PSB and BALF samples were extracted for quantification of bacterial content by RT-qPCR analysis. Six bacterial strains were analyzed using corresponding standard strains as positive controls (Table 5). *Staphylococcus aureus*, *Klebsiella pneumoniae*, *Streptococcus pneumoniae*, *Pseudomonos aeruginosa*, *Haemophilus influenzeae*, and *Moraxella catarrhalis* were recovered from PSB samples in 68.8 %, 46.9 %, 42.8 %, 62.5 %, 78.1 % and 50.0 % of patients in the AECOPD group, respectively; and in 47.1 %, 29.4 %, 58.8 %, 44.1 %, 52.9 % and 64.7 % of patients in the stable COPD group, respectively; compared with 9.1 %, 15.2 %, 30.3 %, 6.1 %, 15.2 % and 12.1 % of HS, respectively (*P* < 0.01). Detected rates of the six strains in BALF samples demonstrated the same diversification trend as those revealed in PSB samples. There was no significant difference in detection rates for the six bacterial strains in BALF samples compared with PSB samples (Table 5).

**Table 5:** RT-qPCR analysis of PSB and BALF samples obtained from patients in the HS, stable COPD and AECOPD groups

***n*** (%)	HS		Stable COPD patients		AECOPD patients	
	(*n* = 33)		(*n* = 34)		(*n* = 32)	
	PSB	BALF	PSB	BALF	PSB	BALF
*Staphylococcus aureus*	3 (9.1)	2 (6.1)	16 (47.1)	17 (50.0)	22 (68.8)	20 (62.5)
*Klebsiella pneumoniae*	5 (15.2)	6 (18.2)	10 (29.4)	9 (26.5)	15 (46.9)	16 (50.0)
*Streptococcus pneumoniae*	10 (30.3)	8 (24.2)	20 (58.8)	18 (52.9)	14 (42.8)	17 (53.1)
*Pseudomonos aeruginosa*	2 (6.1)	2 (6.1)	15 (44.1)	17 (50.0)	20 (62.5)	23 (71.9)
*Haemophilus influenzeae*	5 (15.2)	6 (18.2)	18 (52.9)	20 (58.8)	25 (78.1)	24 (75.0)
*Moraxella catarrhalis*	4 (12.1)	4 (12.1)	22 (64.7)	20 (58.8)	16 (50.0)	15 (46.9)

HS, Healthy subjects

Furthermore, to compare the pathogen detection rate between microbiological culture and RT-qPCR, mean detection rates of the six pathogens by RT-qPCR analysis and conventional microbiological culture from PSB and BALF samples in all subjects were calculated and analyzed. Mean detection rates of *Staphylococcus aureus*, *Klebsiella pneumoniae*, *Streptococcus pneumoniae*, *Pseudomonos aeruginosa*, *Haemophilus influenzeae*, and *Moraxella catarrhalis* by RT-qPCR analysis were 40.6 ± 36.79 %, 31.03 ± 14.5 %, 43.68 ± 13.87 %, 32.62 ± 30.78 %, 49.7 ± 27.28 % and 40.77 ± 23.08 %, respectively; which were significantly higher compared with 7.08 ± 3.68 % (*P* = 0.008), 7.14 ± 4.74 % (*P* = 0.001), 10.07 ± 3.58 % (*P* = 0.0002), 6.15 ± 6.57 % (*P* = 0.022), 8.18 ± 7.84 % (*P* = 0.0018), and 3.07 ± 2.82 % (*P* = 0.0037), respectively, as detected by conventional culture methods.

### Quantification of microorganism load in PSB and BALF samples obtained from COPD patients and HS

**Fig. 1:**
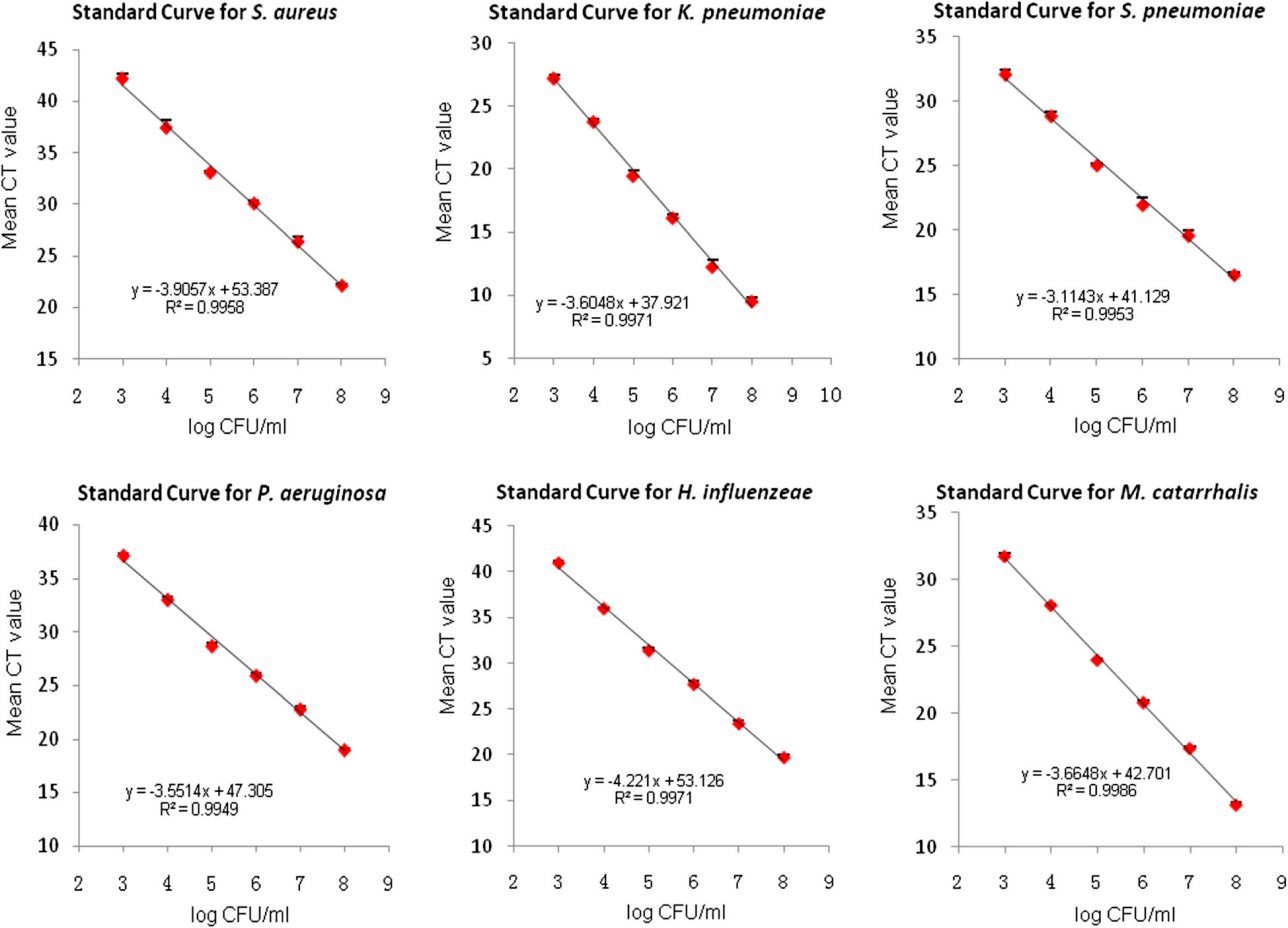
Standard curves for *Staphylococcus aureus*, *Klebsiella pneumoniae*, *Streptococcus pneumoniae*, *Pseudomonos aeruginosa*, *Haemophilus influenzeae*, and *Moraxella catarrhalis* assays. Mean threshold cycle (CT) value ± SEM of three replicates (three per run) for each reaction is shown in relation to the log of CFU calculated per 20 μl of reaction mixture. Relative correlation coefficient (*R* ^*2*^) and linear regression equations are reported in the legend

**Fig. 2:**
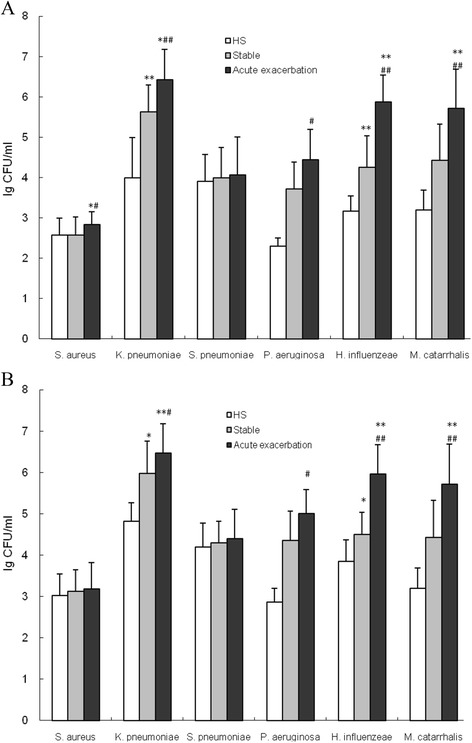
Bacterial load in PSB and BALF samples obtained from COPD patients and healthy subjects. The burden of *Staphylococcus aureus*, *Klebsiella pneumoniae*, *Pseudomonos aeruginosa*, *Haemophilus influenzeae*, and *Moraxella catarrhalis* in PSB samples (**a**), and of *Klebsiella pneumoniae*, *Pseudomonos aeruginosa*, *Haemophilus influenzeae*, and *Moraxella catarrhalis* in BALF samples (**b**) obtained from AECOPD patients compared with stable COPD patients. All data are expressed as means ± SEM. ^*^ *P* < 0.05 and ^**^ *P* < 0.01 *vs*. the HS group; ^#^ *P* < 0.05 and ^##^ *P* < 0.01 *vs*. the stable COPD group

To quantify the number of bacterial cells in PSB and protected BALF samples obtained from patients in the three groups, six ATCC standard strains (*Staphylococcus aureus*, *Klebsiella pneumoniae*, *Streptococcus pneumoniae*, *Pseudomonos aeruginosa*, *Haemophilus influenzeae*, and *Moraxella catarrhalis*) were used as positive controls to correlate CFU with the CT values measured by RT-qPCR. CT values revealed a linear correlation with the 10-log of CFU per ml of bacteria. Therefore, the load of pathogens in PSB and BALF samples were calculated according to a standard curve equation (Fig. 1). A significant increase in the load of *Staphylococcus aureus*, *Klebsiella pneumoniae*, *Pseudomonos aeruginosa*, *Haemophilus influenzeae*, and *Moraxella catarrhalis* in PSB samples obtained from AECOPD patients were detected, as compared with PSB samples obtained from stable COPD patients (Fig. 2a, *P* < 0.01 or *P* < 0.05). The load of *Klebsiella pneumoniae* and *Haemophilus influenzea* in PSB samples obtained from AECOPD patients significantly increased compared with PSB samples obtained from the HS group (*P* < 0.01). However, there was no significant difference in the load of *Streptococcus pneumoniae* between patients in the COPD and HS groups (*P* > 0.05).

There was a significant increase in the load of *Klebsiella pneumoniae*, *Pseudomonos aeruginosa*, *Haemophilus influenzeae*, and *Moraxella catarrhalis* in BALF samples obtained from AECOPD patients compared with BALF samples obtained from stable COPD patients (Fig. 2b, *P* < 0.01 or *P* < 0.05). Moreover, the load of *Haemophilus influenzea* in BALF samples obtained from AECOPD patients significantly increased, compared with BALF samples obtained from patients in the HS group (*P* < 0.05). However, there was no significant difference in the load of *Staphylococcus aureus* and *Streptococcus pneumoniae* between patients in the COPD and HS groups (*P* > 0.05).

### Total and differential cell counts in BALF samples obtained from COPD patients and HS

**Fig. 3:**
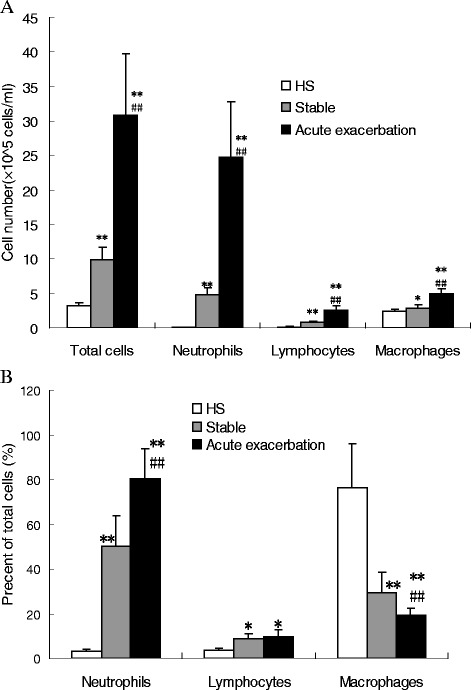
Infiltration of inflammatory cells in airways of COPD patients. The number of total inflammatory cells including neutrophils, lymphocytes, and macrophages in BALF samples obtained from patients in the HS, stable COPD and AECOPD groups were determined (**a**). The percentage of each cell type from total cells (**b**). Data are expressed as means ± SEM (*n* = 20 per group). ^*^ *P* < 0.05 and ^**^ *P* < 0.01 *vs*. the HS group; ^#^ *P* < 0.05 and ^##^ *P* < 0.01 *vs*. the stable COPD group

To assess the degree of lung inflammation in COPD patients, BALF samples were collected; then, total and differential cell counts were analyzed. The total number of alveolar inflammatory cells dramatically increased in AECOPD patients compared with stable COPD patients and HS, and in stable COPD patients compared with HS (*P* < 0.01, Fig. 3a).

The differential cell count of BALF revealed a significant increase in all inflammatory cells in the alveolar space in AECOPD patients compared with stable COPD patients and HS (Fig. 3b). The highest increment was observed in the neutrophil population (*P* < 0.01), but the number of lymphocytes and macrophages also significantly increased in AECOPD patients (*P* < 0.01). However, the percentage of macrophages in the total cell population significantly decreased (*P* < 0.01). The percentage of lymphocytes in stable COPD and AECOPD patients significantly increased compared with HS (*P* < 0.01, Fig. 3b).

### Cytokine levels in BALF supernatants obtained from COPD patients and HS

In order to determine changes in pro-inflammatory cytokines, BALF supernatants obtained from COPD patients and HS were collected. IL-1β, IL-6, IL-8, IL-10 and TNF-α were measured by ELISA. Levels of IL-1β, IL-6, IL-8, IL-10 and TNF-α in BALF supernatants were significantly higher in stable COPD and AECOPD patients compared with HS (Table 6, *P* < 0.01). Moreover, significant differences were observed between stable COPD and AECOPD patients for all cytokines (*P* < 0.01).

**Table 6:** Cytokine levels in BALF supernatants obtained from patients in the HS, stable COPD and AECOPD groups

Cytokines (pg/ml)	HS	Stable COPD patients	AECOPD patients
	(*n* = 33)	(*n* = 34)	(*n* = 32)
IL-1β	0.15 ± 0.05	0.21 ± 0.07^**^	0.58 ± 0.13^**##^
IL-6	0.64 ± 0.15	1.05 ± 0.21^**^	2.07 ± 0.22^**##^
IL-8	6.81 ± 1.59	9.50 ± 1.3^**^	31.39 ± 6.02^**##^
IL-10	0.10 ± 0.06	0.18 ± 0.06^**^	0.30 ± 0.10^**##^
TNF-α	0.02 ± 0.01	0.05 ± 0.01^**^	0.27 ± 0.11^**##^

All data are expressed as means ± SEM. ^*^ *P* < 0.05 and ^**^ *P* < 0.01 *vs*. the HS group; ^#^ *P* < 0.05 and ^##^ *P* < 0.01 *vs*. the stable COPD group. HS, Healthy subjects

### Correlation between bacterial burden, inflammatory index, and pulmonary function of COPD patients and HS

Using *Pearson*’*s* correlation coefficient, positive correlations were detected between the load of *Klebsiella pneumoniae*, *Pseudomonos aeruginosa*, *Haemophilus influenzeae* and *Moraxella catarrhalis*, and inflammatory mediators such as the percentage of neutrophils and concentration of cytokines such as IL-1β, IL-6 and IL-8 in BALF supernatants (Table 7). Moreover, *Pearson*’*s* correlation coefficients between the load of *Staphylococcus aureus*, *Pseudomonos aeruginosa*, *Haemophilus influenzeae* and *Moraxella catarrhalis*, and TNF-α concentrations in BALF supernatants revealed a positive and statistically significant correlation (*P* < 0.01). However, no significant correlation between the load of *Streptococcus pneumoniae* and inflammatory mediators was detected (*P* > 0.05).

**Table 7:** Correlation analysis of bacterial burden and inflammatory index in BALF samples

	*Staphylococcus aureus*		*Klebsiella pneumoniae*		*Streptococcus pneumoniae*		*Pseudomonos aeruginosa*		*Haemophilus influenzeae*		*Moraxella catarrhalis*	
	(log CFU/ml)		(log CFU/ml)		(log CFU/ml)		(log CFU/ml)		(log CFU/ml)		(log CFU/ml)	
	*r*	*p*	*r*	*p*	*r*	*p*	*r*	*p*	*r*	*p*	*r*	*p*
Neutrophil (%)	0.175	0.252	0.458	0.002	0.071	0.641	0.497	0.003	0.564	<0.01	0.551	<0.01
IL-1β (pg/ml)	−0.082	0.511	0.327	0.010	0.086	0.479	0.452	0.001	0.682	<0.01	0.503	<0.01
IL-6 (pg/ml)	0.068	0.589	0.317	0.012	0.082	0.500	0.413	0.004	0.663	<0.01	0.549	<0.01
IL-8 (pg/ml)	0.165	0.187	0.314	0.013	0.044	0.720	0.341	0.018	0.694	<0.01	0.587	<0.01
IL-10 (pg/ml)	−0.042	0.738	0.201	0.118	0.110	0.364	0.205	0.163	0.451	<0.01	0.376	0.003
TNF- α (pg/ml)	0.266	0.031	0.249	0.051	0.121	0.316	0.320	0.027	0.703	<0.01	0.493	<0.01

The correlation between the load of the six pathogens and the FEV1 % predicted, FVC % predicted and FEV1/FVC values were analyzed to assess the relationship between bacterial burden and pulmonary function in the study subjects. *Pearson*’*s* correlation coefficients between the load of *Staphylococcus aureus*, *Klebsiella pneumoniae*, *Streptococcus pneumoniae*, *Pseudomonos aeruginosa*, *Haemophilus influenzeae* and *Moraxella catarrhalis* in PSB samples and the FEV1 % predicted, FVC % predicted, and FEV1/FVC values revealed a negative statistically significant correlation (*P* < 0.01, Table 8).

**Table 8:** Correlation analysis of bacterial burden in PSB samples and pulmonary function

	*Staphylococcus aureus*		*Klebsiella pneumoniae*		*Streptococcus pneumoniae*		*Pseudomonos aeruginosa*		*Haemophilus influenzeae*		*Moraxella catarrhalis*	
	(log CFU/ml)		(log CFU/ml)		(log CFU/ml)		(log CFU/ml)		(log CFU/ml)		(log CFU/ml)	
	*r*	*p*	*r*	*p*	*r*	*p*	*r*	*p*	*r*	*p*	*r*	*p*
FEV1 % predicted	−0.674	<0.01	−0.865	<0.01	−0.543	<0.01	−0.664	<0.01	−0.849	<0.01	−0.813	<0.01
FVC % predicted	−0.597	<0.01	−0.756	<0.01	−0.419	<0.01	−0.586	<0.01	−0.728	<0.01	−0.675	<0.01
FEV1/FVC	−0.660	<0.01	−0.833	<0.01	−0.532	<0.01	−0.639	<0.01	−0.814	<0.01	−0.776	<0.01

## Discussion

Several innate immune mechanisms are involved in maintaining the sterility of a healthy human airway. However, immune mechanisms are disrupted by smoking or other hazardous substances; resulting in the persistence of microbial pathogens in the lower airway of COPD patients [25], which is called “colonization” by some researchers. Previous studies have proven that bacterial colonization is associated with greater levels of airway inflammation measured in sputum [26–28] and has been implicated as the cause of most exacerbations in COPD [29–31]^.^ Acute exacerbations are associated with a more rapid decline in lung function and an impaired quality of life, which are both major causes of morbidity and mortality in COPD [22]. However, the pathogens involved, the number of pathogens that change, and the mechanism on how these infections alter lower airway inflammation remains unclear.

Bacterial colonization and airway inflammation in COPD patients have been previously demonstrated in studies. However, these studies have been limited to sampling the central tracheobronchial tree and/or detecting the pathogen by traditional cultures [26–28]. In this present study, the load of pathogenic bacteria strain *Staphylococcus aureus*, *Klebsiella pneumoniae*, *Streptococcus pneumoniae*, *Pseudomonos aeruginosa*, *Haemophilus influenzeae* and *Moraxella catarrhali* were detected in PSB and BALF samples obtained from COPD patients and HS by RT-q-PCR assays, targeting specific pathogen genes. Moreover, cell count in BALF and pro-inflammatory cytokine concentrations in BALF supernatants were analyzed. Significantly higher levels of *Klebsiella pneumoniae*, *Pseudomonos aeruginosa*, *Haemophilus influenzeae*, and *Moraxella catarrhalis* were identified in both PSB and BALF samples obtained from stable COPD and AECOPD patients. Additionally, a significantly higher number of total cells and percentage of neutrophils in BALF, together with higher levels of IL-1 β, IL-6, IL-8, IL-10 and TNF-α in BALF supernatants, were detected in stable COPD and AECOPD patients. More importantly, pathogen loads in BALF samples were positively correlated with the percentage of neutrophils and levels of pro-inflammatory cytokines in BALF. Pathogen loads in PSB samples were negatively correlated with FEV1 % predicted, FVC % predicted, and FEV1/FVC values. Thus, the increase of pathogens in the lower respiratory tract likely contributed to the inflammatory response in the airways, leading to recurrent AECOPD and pulmonary function decline.

Obtaining samples from the lower respiratory tract without being contaminated by the upper airway flora is a crucial factor in accurately determining the prevalence of bacteria in the lower respiratory tract. Previous studies have found higher rates of bacterial isolates in sputum than in samples collected by bronchoscopy in stable COPD patients [32–34]. This is likely due to the high contamination rate of sputum with the upper respiratory tract flora. Therefore, bronchoscopy with PSB appears as the best method to avoid upper airway contamination. For the same reason, protected BALF samples are appropriate for contamination-free pathogen analysis. Our results did not reveal significant differences in rates of the six analyzed pathogens between PSB and BALF samples among the three study groups. However, significantly higher rates of these six pathogens were found in PSB and BALF samples obtained from COPD patients compared with samples obtained from HS, and no significant differences between stable COPD and AECOPD patients were detected. These data confirms bacterial colonization in the lower respiratory tract of COPD patients, regardless of the patient’s status (stable or acute exacerbation of COPD). Nevertheless, persistent symptoms and recurrent exacerbations were seen in ex-smokers with moderate to severe COPD, suggesting that persistent inflammation resulting from bacterial infections has clinical consequences. Thus, the number of bacteria in the airway is a key factor for the development of AECOPD in stable COPD pateints.

Previous studies have demonstrated bacterial load changes in airways of COPD patients using conventional methods [26–29]. However, conventional methods such as microbiological cultures are time-consuming and may give false-negative results, especially during ongoing antibiotic treatments. Non-culture based methods such as RT-qPCR are sensitive, specific and provide fast results; and are valuable tools for the early diagnosis and effective therapy of COPD. Erb-Downward *et al*. identified a core pulmonary bacterial microbiome that includes *Pseudomonas*, *Streptococcus*, *Prevotella*, *Fusobacterium*, *Haemophilus*, *Veillonella*, and *Porphyromonas* by massively parallel pyrosequencing of bacterial 16S amplicons [35]^.^ In another study, the composition of the lung microbiome was determined using 454 pyrosequencing of 16S rDNA in BALF; and found that the main phyla in all samples were *Actinobacteria*, *Firmicutes*, and *Proteobacteria* [17]. However, analysis of microbiomes by 16S rDNA or 16 s rRNA focuses in building a picture of the complete microbial community in an environment, making a cluster analysis and studying the evolution history of microbiomes. Nevertheless, it is important to identify common bacterial species in the lower respiratory tract of COPD patients for guidance in clinical antibiotic therapy and analysis of inflammatory response. In this present study, RT-qPCR analysis revealed significantly higher detection rates for *Staphylococcus aureus*, *Klebsiella pneumoniae*, *Streptococcus pneumoniae*, *Pseudomonos aeruginosa*, *Haemophilus influenzeae*, and *Moraxella catarrhalis*, compared to conventional microbiological culture in all subjects. These results reflect the higher sensitivity and specificity of RT-qPCR, as a fast pathogen detection method.

Pathogens such as *Streptococcus pneumoniae*, *Haemophilus influenzae*, and *Moraxella catarrhalis* are associated with approximately 50 % of COPD exacerbations, as demonstrated by traditional microbiological culture. These organisms can often be found colonizing the respiratory airways of COPD patients between exacerbations [5], and are consistent with our culture results. However, bacteria in COPD varies with disease severity, as *Pseudomonas aeruginosa* is more commonly detected in patients with severe COPD in both stable [36, 37] and acute exacerbations [38–40]. Microbiome analysis by 16S rDNA or 16 s rRNA also revealed that *Haemophilus* species were strongly associated with the presence of COPD [41], while *Pseudomonas* species were more commonly observed in subjects with moderate or severe COPD [35]. Patients in our present study presented with moderate and severe COPD (GOLD stage II-III), and the load of *Klebsiella pneumoniae*, *Haemophilus influenzeae*, *Moraxella catarrhalis* and *Pseudomonos aeruginosa* in PSB and BALF samples obtained from COPD patients significantly increased compared with samples obtained from HS, as detected by RT-qPCR; suggesting that bacterial species quantitatively analyzed by RT-qPCR are consistent with bacterial species cultured in previous studies. Moreover, the load of *Klebsiella pneumoniae*, *Haemophilus influenzeae*, *Moraxella catarrhalis* and *Pseudomonos aeruginosa* in AECOPD patients also significantly increased compared with stable COPD patients; which suggest that this increase of bacterial load in the lower respiratory tract may contribute to acute exacerbation in COPD. A previous study has indicated the colonization of *Streptococcus pneumoniae* in AECOPD patients [42]. In this present study, there was no significant increase of *Streptococcus pneumoniae* in PSB and BALF samples from AECOPD patients, compared with stable COPD patients and HS. However, our study results had no discrepancy with previous studies, because high loads of *Streptococcus pneumoniae* were found in both stable COPD patients and HS; which is consistent with a previous study [35]. Our results by RT-qPCR indicated that *Streptococcus pneumoniae* was present in all study subjects, although previous studies did not detect a high load of *Streptococcus pneumoniae* in these subjects by using conventional culture methods.

In the contrary, diagnosing lower respiratory tract infections caused by *Streptococcus peumoniae*, *Haemophilus influenzae*, and *Moraxella catarrhalis* by PCR has been limited to distinguishing colonization from infections. This may depend on the analysis that combined the load of the above pathogens with symptoms and inflammatory mediators such as C-reactive protein, the number of neutrophils, and some pro-inflammatory cytokines. Exacerbations are typically associated with increased neutrophilic airway inflammation [43, 44]. Pro-inflammatory cytokines such as TNF-α, IL-1 β and IL-6 are increased in COPD and appear to amplify inflammation [45]. In this present study, there was a significant increase in the number of neutrophils in BALF, and in the levels of pro-inflammatory cytokines IL-1 β, IL-6, IL-8, IL-10 and TNF-α in BALF supernatants obtained from AECOPD patients, compared with stable COPD patients and HS; and there was a positive correlation between the load of *Klebsiella pneumoniae*, *Haemophilus influenzeae*, *Moraxella catarrhalis* and *Pseudomonos aeruginosa*, and most inflammatory mediators. These data indicate that the increase in the load of common pathogens in the lower respiratory tract of COPD patients may contribute to the increase in pro-inflammatory response as acute exacerbations occur, resulting in disease progression and gradual decline in lung function. Indeed, there was a negative correlation between the load of the six pathogens and the FEV1 % predicted, FVC % predicted and FEV1/FVC values, which revealed that increased loads of common pathogens led to the decline of lung functions in COPD patients. However, in this present study, there was no quantitative data regarding changes in the above bacteria in the lower respiratory tract of mild COPD patients (GOLD stage I). Thus, the association between the load of these six bacteria and the degree of airflow obstruction in COPD patients was limited, requiring a more in-depth study in the future.

## Conclusions

In summary, our data indicate that significantly higher loads of *Klebsiella pneumoniae*, *Haemophilus influenzeae*, *Moraxella catarrhalis*, and *Pseudomonos aeruginosa* exist in PSB and BALF samples obtained from AECOPD patients, compared with stable COPD patients and HS, as revealed by RT-qPCR. Higher loads were correlated with increased inflammatory response in the lower respiratory tract, and were associated with disease progression and decline in lung function in COPD patients. Our study may provide a new standard for the quantitative measurement of common bacteria in the lower respiratory tract of moderate and severe COPD patients, and provide some guidance in determining the most appropriate antibiotic therapy for AECOPD patients. Limitations of this study include small sample size, lack of healthy smokers and mild COPD patients, as well as the relationship between bacterial load and immune barrier of the lower respiratory tract in COPD patients. Therefore, further studies with a larger patient population are warranted to address these issues.

## Abbreviations

COPD: Chronic obstructive pulmonary disease
RT-qPCR: Real-time quantitative PCR
PSB: Protected specimen brush
BALF: Bronchoalveolar lavage fluid
IL: Interleukin
ELISA: Enzyme-linked immunosorbent assay
DGGE: Denaturing gradient gel electrophoresis
GOLD: Global initiative for chronic obstructive lung disease
HS: Healthy Subjects
FEV1: Forced expiratory volume in one second
FVC: Forced vital capacity
TNF: Tumor neurosis factor
CT: Cycle threshold
CRP: C-reactive protein
